# Supplementary material for: Ancient mitochondrial DNA pathogenic variants putatively associated with mitochondrial disease
Source: PLoS One. 2020 Sep 24;15(9):e0233666. doi: 10.1371/journal.pone.0233666 (PMC7514063; doi:10.1371/journal.pone.0233666)
Supplement: Attachment — Submitted filename: Response to Reviewers.doc [file pone.0233666.s001.doc]

*Dear Sirs,*

*Thank you for reviewing our manuscript and for letting us resubmit a revised version. We find all comments to be very helpful and constructive. We have taken up your suggestions, and the manuscript has been thoroughly revised. Notably, following the advice of the reviewer, we now discuss the spatiotemporal dynamics of the putatively pathogenic variants established in ancient samples based on their geographic location, contemporary epidemiological trends and haplogroup affiliation. Below, we explain in detail how we have addressed the various comments and suggestions. We believe that these changes have resulted in a much better version of the manuscript, which we hope is more befitting for publication in PLOS ONE.*

*Sincerely,*

*Prof. Draga Toncheva*

Academic editor`s comments:

1. Please ensure that your manuscript meets PLOS ONE's style requirements, including those for file naming.

***Our Reply:*** *We have acquainted ourselves with* PLOS *ONE style templates and made sure the manuscript meets the journal`s style requirements*

2. PLOS requires an ORCID iD for the corresponding author in Editorial Manager on papers submitted after December 6th, 2016. Please ensure that you have an ORCID iD and that it is validated in Editorial Manager.

***Our Reply:*** *We updated the corresponding author`s information in Editorial Manager and validated the ORCID iD.*

3. We note that Figure 1 in your submission contain [map/satellite] images which may be copyrighted. All PLOS content is published under the Creative Commons Attribution License (CC BY 4.0), which means that the manuscript, images, and Supporting Information files will be freely available online, and any third party is permitted to access, download, copy, distribute, and use these materials in any way, even commercially, with proper attribution. For these reasons, we cannot publish previously copyrighted maps or satellite images created using proprietary data, such as Google software (Google Maps, Street View, and Earth). For more information, see our copyright guidelines: http://journals.plos.org/plosone/s/licenses-and-copyright.

***Our reply:*** *We have now replaced this with our own very basic image that we deem sufficient.*

REVIEWER(S)' COMMENTS:

Reviewer #1: In the present paper, authors investigated on pathogenic mutations in ancient mtDNA, providing further insight into the emergence of mitochondrial diseases.

Although not all available ancient mitogenomes were considered, sources were reported and the analyses were detailed and appropriate. I have minor concerns regarding this study as it is currently presented and discussed.

The authors could make modifications throughout the text to increase the quality of the manuscript and to comply with the standards for publication in Plos One.

***Our Reply:*** *We have thoroughly revised our manuscript to meet the standards for publication in PLOS ONE.*

I suggest authors to improve their discussion by exploring the distribution of the mitochondrial pathogenic variants in modern populations, their geographic distribution and/or their differential distribution in the mitochondrial haplogroups. The latter could be an interesting discussion if compared with the mitochondrial haplogroup found in ancient samples harboring the same pathogenic mutations.

***Our Reply:*** *We have now added additional text at the beginning of the Discussion section exploring the overlap in distribution of the pathogenic variants in ancient and modern populations, and their differential distribution in the mitochondrial haplogroups.*

Few minor points to be checked are the following:

Line 44 “Exposing pathogenic variants in ancient human populations expands our understanding of their origin”: Authors could better argue this sentence in the conclusion paragraph and further explain the importance of this study.

***Our Reply:*** *We have now added similar sentence in the “Conclusion” paragraph*

Line 115 “confirmed pathogenic|” should modified in “confirmed pathogenic”

***Our Reply:*** *We have corrected this.*

Line 135 “mitochondrial myopathy and ophtalmoplegia (MM)” MM is for mitochondrial myopathy thus I suggest to modify as follows: “mitochondrial myopathy (MM) and ophtalmoplegia”.

***Our Reply:*** *We have corrected this.*

Line 144 “Population-based studies suggest the m.3243A>G mutation is the most common disease-causing…” Sentence has something wrong, please verify and change.

***Our Reply:*** *The phrase “the most common disease-causing” is changed to “one of the most common disease-causing”, and a reference is added to substantiate this claim.*

Line 88: “We used various publicly available databases to gather information to identify mtDNA sequence variants and to gather information about each variant…” Sentence has too much repetitions, please rephrase.

***Our Reply:*** *This sentence has now been changed to “*We used various publicly available databases to collect information on mtDNA sequence variants, including its clinical significance and contemporary population frequencies”

Line 161 I would prefer McFarland and colleagues instead of McFarland et al.

***Our Reply:*** *We have now changed that.*

Line 175 Gill et al. Instead of Gill et al

The same for Houshmand et al line 187, and line 202 where “et al” should be “et al.“

***Our Reply:*** *We have now corrected these.*

Line 188-190. Sentence is confusing. Please rephrase

***Our Reply:*** *You are absolutely right! We have now changed this sentence to “Nucleotides that are conserved between species are unaffected by this mutation, and the authors presume that it is unlikely to be pathogenic.”*

Table 1: notes are unclear because they are covered by row numbering. If I am right MERRF and

MEPR acronyms are reported twice.

***Our Reply:*** *We have now corrected this.*

Authors consider samples dated before 375CE as Middle Ages as reported in AmtDB database. As they belong to the Roman period, I think that this historical classification is arguable.

***Our Reply:*** *We have used the annotation given in the Ancient mtDNA database (Ehler E, et al. AmtDB: a database of ancient human mitochondrial genomes. Nucleic acids research. 2018;47), where these samples were taken from.*

Line 206-209: “Likely/possibly pathogenic mutations” I suggest authors to briefly describe in this paragraph the main results even if they are summarized in the table.

***Our Reply:*** *We have now added that they are ten mutations. We choose to concentrate our attention to the group of “confirmed pathogenic” mutations and feel additional mention of these results is unnecessary.*

Line 275: “Model 0 model” the word model is present twice.

***Our Reply:*** *We have now corrected this.*

Line 365 “…mutation in the mitochondrial tRNA <sup>Leu(UUR)</sup> gene. Diabetes. 1994;43(6):746”. Please change in “…mutation in the mitochondrial tRNA(Leu(UUR)) gene. Diabetes. 1994;43(6):746‐751”.

***Our Reply:*** *We have now corrected this.*
